# Supplementary material for: Modelling the climatic suitability of Chagas disease vectors on a global scale
Source: eLife. 2020 May 6;9:e52072. doi: 10.7554/eLife.52072 (PMC7237218; doi:10.7554/eLife.52072)
Supplement: Source code 1. [file elife-52072-code1.docx]

Source code File 1: Source code for modelling the climatic suitability of Chagas disease vectors on a global scale. Modelling was executed in the R environment (version 3.6.0) using the biomod2 package (version 3.3-7.1).

############################### Occurrence data ###################################

install.packages("maptools")

install.packages("SDMTools")

library(sp);

library(rgdal);

library(raster);

library(maptools);

library(SDMTools);

library(raster);

library(biomod2)

setwd("C:/Path/To/Occurrence/Data")

species <- c("P.geniculatus","P.megistus","R.brethesi", "R.ecuadoriensis","R.prolixus","T.brasiliensis",

"T.dimidiata","T.infestans","T.maculata","T.rubrofasciata","T.sordida")

i=1

sp_name <- species[i]

sp_name

# an asc-file of the environmental variables is used as a mask for the spatial reference

aa <- read.asc("C:/Path/To/Environmental/Variables/bio04.asc")

mask <- raster.from.asc(aa)

# occurrence data with correct spatial reference (structure: species, lon, lat)

occ <- read.csv(paste ("C:/Path/To/Occurrence/Data/Files/occ_",sp_name,".csv", sep=""))

occ_ <- occ[,2:3]

r <- raster.from.asc(aa)

xy <- occ_

x <- rasterize(xy, r, fun='count')

x <- 1 *(x > 0)

tt <- data.frame(coordinates(x),count=x[])

colnames(tt) <- c("lon", "lat", sp_name)

for (i in 2:11)

{

sp_name <- species[i]

print(sp_name)

occ <- read.csv(paste ("C:/Path/To/Occurrence/Data/Files/occ_",sp_name,".csv", sep=""))

str(occ)

points(occ[,2],occ[,3], pch=20, col="green")

xy <- occ[,2:3]

x <- rasterize(xy, r, fun='count')

x <- 1 *(x > 0)

tt <-cbind(tt,count=x[])

}

colnames(tt) <- c("lon", "lat", species[1:11]) #colnames(tt) <- c("lon", "lat", species[1:xxxx])

write.csv(tt, "occ_data_as_table_biomod.csv")

sp.names <- c("P.geniculatus","P.megistus","R.brethesi","R.ecuadoriensis","R.prolixus", "T.brasiliensis", "T.dimidiata","T.infestans","T.maculata","T.rubrofasciata","T.sordida")

mySpeciesOcc <- read.csv("occ_data_as_table_biomod.csv")

################################## AUC values #####################################

#initialise matrix

AUC_matrix <- matrix(data=NA, nrow=length(sp.names), ncol=16)

colnames(AUC_matrix) <- c("Species",'GLM1','GBM1','GAM1','ANN1','MARS1','GLM2','GBM2','GAM2','ANN2','MARS2','GLM3','GBM3','GAM3','ANN3','MARS3')

output.dir <- "C:/Path/To/Output/Directory/output_dir"

############################### Environmental data #################################

env.dir <- " C:/Path/To/Environmental/Variables/Of/South/America/"

l <- list.files(env.dir, pattern= ".asc")

myExpl = stack( raster(paste(env.dir, "bio04.asc",sep="")),

raster(paste(env.dir, "bio05.asc",sep="")),

raster(paste(env.dir, "bio06.asc",sep="")),

raster(paste(env.dir, "bio13.asc",sep="")),

raster(paste(env.dir, "bio14.asc",sep="")),

raster(paste(env.dir, "bio15.asc",sep="")))

plot(myExpl)

# correlation

env <- as(myExpl,"SpatialGridDataFrame")

env <- as.data.frame(env)

cor_matrix <- cor(env,use = "complete.obs", method = "pearson" )

write.csv(cor_matrix , "cor_matrix_pearson.csv")

myExpl_current <- myExpl

################################## Modelling options #################################

myBiomodOptions <- BIOMOD_ModelingOptions(

GLM = list( test = 'AIC'),

GBM = list( n.trees = 5000,

interaction.depth = 7,

n.minobsinnode = 10,

shrinkage = 0.01),

GAM = list( algo = 'GAM_mgcv',

type = 's_smoother',

k = 4,

family = binomial(link = 'logit'),

method = 'GCV.Cp'),

ANN = list( size = 8,

decay = 0.001),

MARS = list( penalty = 1,

nprune = 17),

MAXENT.Phillips = list( path_to_maxent.jar = getwd(),

maximumiterations = 10000,

visible = FALSE,

linear = TRUE,

quadratic = TRUE,

product = TRUE,

threshold = F,

hinge = F,

lq2lqptthreshold = 80,

l2lqthreshold = 10,

hingethreshold = 15,

beta_threshold = -1,

beta_categorical = -1,

beta_lqp = -1,

beta_hinge = -1,

defaultprevalence = 0.5))

################################### Modelling ######################################

i=1

sp.n = paste(sp.names[i])

sp.n

cat('\n',sp.n,'modeling...')

myResp <- as.numeric(mySpeciesOcc[,sp.n])

na.id <- which(is.na(myResp))

# remove NAs to force the pseudo-absence extraction from background data

if (length(na.id)!=0)

{myResp <- myResp[-na.id]} ## presence-only data

myRespCoord = mySpeciesOcc[,c('lon','lat')] ## coordinates of the presence-only data

if (length(na.id)!=0)

{myRespCoord <- myRespCoord[-na.id,]}

myRespName = sp.n

myBiomodData <- BIOMOD_FormatingData(resp.var = myResp,

expl.var = myExpl,

resp.xy = myRespCoord,

resp.name = myRespName,

PA.nb.rep = 1,

PA.nb.absences = 10000,

PA.strategy = 'disk',

PA.dist.min = 80000)

myBiomodModelOut <- BIOMOD_Modeling(myBiomodData,

models = c('GLM', 'GBM', 'GAM', 'ANN', 'MARS'),

models.options = myBiomodOptions,

NbRunEval=3,

DataSplit=70,

Prevalence=0.5,

VarImport=3,

models.eval.meth = c('KAPPA','TSS','ROC'),

SaveObj = TRUE,

rescale.all.models = TRUE,

do.full.models = FALSE,

modeling.id = paste(myRespName,"_run5",sep=""))

######################### AUC values and variable importance ###########################

ev_e <- get_evaluations(myBiomodModelOut)

write.csv(ev_e,

paste(output.dir,myRespName,"_evaluation_singleModels_disk",".csv", sep=""))

AUC_values <- ev_e[3,1,,,1]

AUC_matrix[i,1] <- paste(sp.names[i])

AUC_matrix[i,2:16] <- AUC_values

var_imp <- myBiomodModelOut@variables.importances@val[,,1,1]

write.csv(var_imp, paste(output.dir,

myRespName,"_var_imp_disk",".csv", sep=""))

############################ Building ensemble models ################################

myBiomodEM <- BIOMOD_EnsembleModeling(

modeling.output = myBiomodModelOut,

chosen.models = 'all',

em.by='all',

eval.metric = c('ROC'),

eval.metric.quality.threshold = c(0.8),

prob.mean = T,

prob.cv = T,

prob.ci = T,

prob.ci.alpha = 0.05,

prob.median = T,

committee.averaging = T,

prob.mean.weight = T,

prob.mean.weight.decay = 'proportional' )

myBiomodEM

# get evaluation scores

ev <- get_evaluations(myBiomodEM)

ev <- ev[[7]]

write.csv(ev,

paste(output.dir,myRespName,"_evaluation_EnsembleModel_disk",".csv", sep=""))

############################# Projection of the models ################################

env.dir.global <- " C:/Path/To/Global/Environmental/Variables/"

l.global <- list.files(env.dir.global, pattern= ".asc")

myExpl.global = stack( raster(paste(env.dir.global, "bio04.asc",sep="")),

raster(paste(env.dir.global, "bio05.asc",sep="")),

raster(paste(env.dir.global, "bio06.asc",sep="")),

raster(paste(env.dir.global, "bio13.asc",sep="")),

raster(paste(env.dir.global, "bio14.asc",sep="")),

raster(paste(env.dir.global, "bio15.asc",sep="")))

plot(myExpl.global)

myExpl_current.global <- myExpl.global

myBiomodProj <- BIOMOD_Projection(

modeling.output = myBiomodModelOut,

new.env = get(paste("myExpl.global", sep="" )),

proj.name = paste("ModelProjections"),

selected.models = 'all',

binary.meth= 'ROC',

compress = 'xz',

clamping.mask = T)

setwd("C:/Path/To/Occurrence/Data ")

png(filename = paste(output.dir,myRespName,"_single_models_disk",".png", sep=""),

width = 960, height = 1140, units = "px", pointsize = 12)

plot(myBiomodProj) #plottet die karte in die png-Datei

dev.off()

####################### Projection of ensemble forecast models ##########################

myBiomodEF <- BIOMOD_EnsembleForecasting(

EM.output = myBiomodEM,

projection.output = myBiomodProj,

#get(paste("myExpl_",Zeiten[z], sep="" )),

#xy.new.env = myRespXY,

selected.models = 'all',

proj.name = paste("ModelEnsembleForecast"),

binary.meth = 'ROC',

filtered.meth = NULL,

compress = T,

build.clamping.mask = TRUE)

png(filename = paste(output.dir,myRespName,"_ensemble_models_disk",".png", sep=""),

width = 960, height = 960, units = "px", pointsize = 12)

plot(myBiomodEF)

dev.off()

################################## Write ascii-files ###################################

dir.create(paste("C:/Path/To/Output/Directory/",sp.names[i],"/asc/",sep=""))

out.dir <- paste("C:/Path/To/Output/Directory/",sp.names[i],"/asc/",sep="")

# single models

C <- paste("C:/Path/To/Output/Directory/",sp.names[i],

"/proj_",'ModelProjections',"/proj_",'ModelProjections', "_",sp.names[i], ".grd", sep="")

D <- paste("C:/Path/To/Output/Directory/",sp.names[i],

"/proj_",'ModelProjections',"/proj_",'ModelProjections', "_",sp.names[i], "_ROCbin.grd", sep="")

A1 =stack(C)[[1]]

name <- names(A1)

A1 <- A1/1000

writeRaster(A1, paste(out.dir,name,"_", ".asc", sep=""))

A2 =stack(C)[[2]]

name <- names(A2)

A2 <- A2/1000

writeRaster(A2, paste(out.dir,name,"_", ".asc", sep=""))

A3 =stack(C)[[3]]

name <- names(A3)

A3 <- A3/1000

writeRaster(A3, paste(out.dir,name,"_", ".asc", sep=""))

A4 =stack(C)[[4]]

name <- names(A4)

A4 <- A4/1000

writeRaster(A4, paste(out.dir,name,"_", ".asc", sep=""))

A5 =stack(C)[[5]]

name <- names(A5)

A5 <- A5/1000

writeRaster(A5, paste(out.dir,name,"_", ".asc", sep=""))

B1 =stack(D)[[1]]

name <- names(A1)

B1 <- B1/1000

writeRaster(B1, paste(out.dir,name,"_", "_ROC.asc", sep=""))

B2 =stack(D)[[2]]

name <- names(A2)

B2 <- B2/1000

writeRaster(B2, paste(out.dir,name,"_", "_ROC.asc", sep=""))

B3 =stack(D)[[3]]

name <- names(A3)

B3 <- B3/1000

writeRaster(B3, paste(out.dir,name,"_", "_ROC.asc", sep=""))

B4 =stack(D)[[4]]

name <- names(A4)

B4 <- B4/1000

writeRaster(B4, paste(out.dir,name,"_", "_ROC.asc", sep=""))

B5 =stack(D)[[5]]

name <- names(A5)

B5 <- B5/1000

writeRaster(B5, paste(out.dir,name,"_", "_ROC.asc", sep=""))

# Ensemble forecasting models

dir.create(paste("C:/Path/To/Output/Directory/",sp.names[i],"/asc_EF/",sep=""))

out.dir <- paste("C:/Path/To/Output/Directory/",sp.names[i],"/asc_EF/",sep="")

EM <- paste("C:/Path/To/Output/Directory/",sp.names[i],

"/proj_",'ModelEnsembleForecast',"/proj_",'ModelEnsembleForecast', "_",sp.names[i], "_","ensemble.grd", sep="")

EM_d <- paste("C:/Path/To/Output/Directory/",sp.names[i],

"/proj_",'ModelEnsembleForecast',"/proj_",'ModelEnsembleForecast', "_",sp.names[i], "_","ensemble_ROCbin.grd", sep="")

A =stack(EM)[[7]]

name <- names(A)

A <- A/1000

writeRaster(A, paste(out.dir,name,"_", ".asc", sep=""))

A =stack(EM_d)[[7]]

name <- names(A)

A <- A/1000

writeRaster(A, paste(out.dir,name,"_",sp.names[i], "_ROC.asc", sep=""))

#################### Export clamping mask, convert grd-file to ascii-file #####################

library(sp);

library(rgdal);

library(raster);

library(maptools);

library(SDMTools);

library(raster);

library(biomod2)

dir.create(paste("C:/Path/To/Output/Directory/asc_EF_clamping_mask/",sep=""))

out.dir <- paste("C:/Path/To/Output/Directory/asc_EF_clamping_mask/",sep="")

species <- c("P.geniculatus","P.megistus","R.brethesi", "R.ecuadoriensis","R.prolixus", "T.brasiliensis","T.dimidiata","T.infestans","T.maculata","T.rubrofasciata","T.sordida")

i=1

sp.names <- species[i]

sp.names

EM <- paste("C:/Path/To/Model/Output/",sp.names,"/proj_ModelProjections/proj_ModelProjections_ClampingMask.grd",sep = "")

A =stack(EM)[[1]]

name <- names(A)

A <- A/1000

writeRaster(A, paste(out.dir,name,"_",sp.names,"_","ClampingMask", ".asc", sep=""))

png(filename = paste(out.dir,name,"_",sp.names,"_","ClampingMask", ".png", sep=""),

width = 960, height = 960, units = "px", pointsize = 12)

plot(A)

dev.off()

######################### Occurrence records per species ###############################

a <- read.csv("occ_data_as_table_biomod.csv")

library(plyr)

count(a, "P.geniculatus")

################## Normalise variable importance for comparison #########################

install.packages("BBmisc")

library(BBmisc)

species <- c("P.geniculatus","P.megistus","R.brethesi", "R.ecuadoriensis","R.prolixus", "T.brasiliensis","T.dimidiata","T.infestans","T.maculata","T.rubrofasciata","T.sordida")

i=1

sp.names <- species[i]

sp.names

v <- read.csv(paste("output_dir",sp.names,"_var_imp.csv",sep=""))

normalized_V <- normalize(v, method = "range", range = c(0, 1), margin = 2, on.constant = "quiet")

write.csv(normalized_V,paste("C:/Path/To/Output/Directory/",sp.names,"/var_imp_normalized.csv",sep=""), row.names = TRUE)

k <- read.csv(paste("var_imp_normalized.csv",sep=""))

# bind all metrics in a one table

z <- bind_rows(list(a,b,c,d,e,f,g,h,x,j,k), .id="id")

write.csv(z,paste("C:/Path/To/Ouput/Directory/var_imp_normalized_all_species.csv",sep=""), row.names = TRUE)
