# Supplementary material for: Modelling the climatic suitability of Chagas disease vectors on a global scale
Source: eLife. 2020 May 6;9:e52072. doi: 10.7554/eLife.52072 (PMC7237218; doi:10.7554/eLife.52072)
Supplement: Supplementary file 1. [file elife-52072-supp1.docx]

Supplementary File 1: Modelled climatic suitability [%] for all occurrences of *T. rubrofasciata* outside of the Americas.

| **Modelled climatic suitability [%]** | **Country** |
| --- | --- |
| 86.3 | Mauritius |
| 83.9 | China |
| 78,5 | Hawaii |
| 79.2 | China |
| 74.9 | China |
| 73 | China |
| 73 | China |
| 72.9 | China |
| 67.7 | Taiwan |
| 66 | China |
| 64.3 | Vietnam |
| 52.9 | Vietnam |
| 52.9 | Philippines |
| 45.9 | India |
| 45.1 | Andaman Islands |
| 44.5 | Philippines |
| 42.6 | India |
| 34.8 | Japan |
| 18.1 | Japan |
| 8.4 | Japan |
| 1.3 | Singapore |
