# Supplementary material for: Modelling the climatic suitability of Chagas disease vectors on a global scale
Source: eLife. 2020 May 6;9:e52072. doi: 10.7554/eLife.52072 (PMC7237218; doi:10.7554/eLife.52072)
Supplement: Supplementary file 2. [file elife-52072-supp2.docx]

Supplementary File 2: AUC values of all algorithms for all considered species.

| **Species** | **AUC value of single algorithms** | | | | | |
| --- | --- | --- | --- | --- | --- | --- |
|  | **ANN** | **GAM** | **GBM** | **GLM** | **MARS** | **Maxent** |
| *P. geniculatus* | 0.932 | 0.96 | 0.984 | 0.95 | 0.963 | 0.663 |
| *P. megistus* | 0.847 | 0.921 | 0.975 | 0.897 | 0.928 | 0.83 |
| *R. brethesi* | 0.898 | 0.959 | 0.961 | 0.955 | 0.951 | 0.928 |
| *R. ecuadoriensis* | 0.838 | 0.854 | 0.916 | 0.928 | 0.829 | 0.954 |
| *R. prolixus* | 0.927 | 0.944 | 0.978 | 0.939 | 0.946 | 0.825 |
| *T. brasiliensis* | 0.889 | 0.975 | 0.992 | 0.962 | 0.978 | 0.935 |
| *T. dimidiata* | 0.79 | 0.841 | 0.922 | 0.819 | 0.861 | 0.817 |
| *T. infestans* | 0.822 | 0.917 | 0.975 | 0.888 | 0.928 | 0.762 |
| *T. maculata* | 0.893 | 0.942 | 0.962 | 0.945 | 0.945 | 0.908 |
| *T. rubrofasciata* | 0.767 | 0.81 | 0.939 | 0.791 | 0.855 | 0.8 |
| *T. sordida* | 0.858 | 0.909 | 0.98 | 0.9 | 0.926 | 0.835 |
