# Supplementary material for: Modelling the climatic suitability of Chagas disease vectors on a global scale
Source: eLife. 2020 May 6;9:e52072. doi: 10.7554/eLife.52072 (PMC7237218; doi:10.7554/eLife.52072)
Supplement: Supplementary file 3. [file elife-52072-supp3.docx]

Supplementary File 3: Sensitivity and specificity metrics of the South American training dataset for all considered species and the independent global sensitivity analysis of *T. rubrofasciata*.

| **Species**  **(Cross-validation)** | **Sensitivity** | **Specificity** |
| --- | --- | --- |
| *Panstrongylus geniculatus* | 93.2 | 93.4 |
| *P. megistus* | 84.4 | 96.1 |
| *Rhodnius brethesi* | 100 | 92.77 |
| *R. ecuadoriensis* | 93.8 | 99.1 |
| *R. prolixus* | 92.9 | 92.4 |
| *Triatoma brasiliensis* | 96 | 95.6 |
| *T. dimidiata* | 89.7 | 90 |
| *T. infestans* | 86.2 | 96.4 |
| *T. maculata* | 97.5 | 93.9 |
| *T. rubrofasciata* | 91.8 | 93.2 |
| *T. sordida* | 92.4 | 89.4 |
| **Species**  **(Global validation)** | **Sensitivity** | **Specificity** |
| *T. rubrofasciata* | 61.9 | - |
