# Supplementary figures and images for: Modelling the climatic suitability of Chagas disease vectors on a global scale
Source: eLife. 2020 May 6;9:e52072. doi: 10.7554/eLife.52072 (PMC7237218; doi:10.7554/eLife.52072)

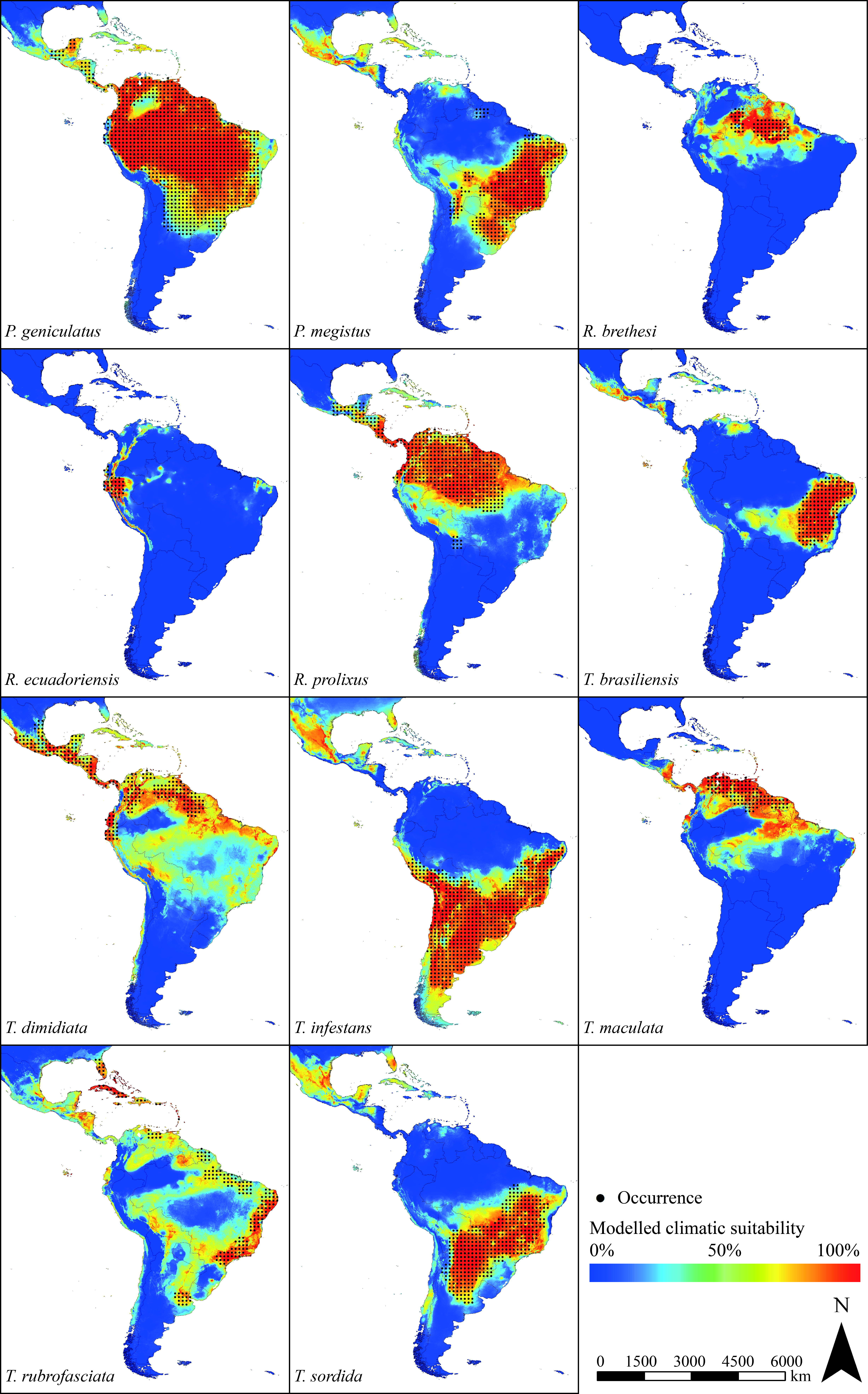

Supplement: Supplementary file 4. [file elife-52072-supp4.jpg]

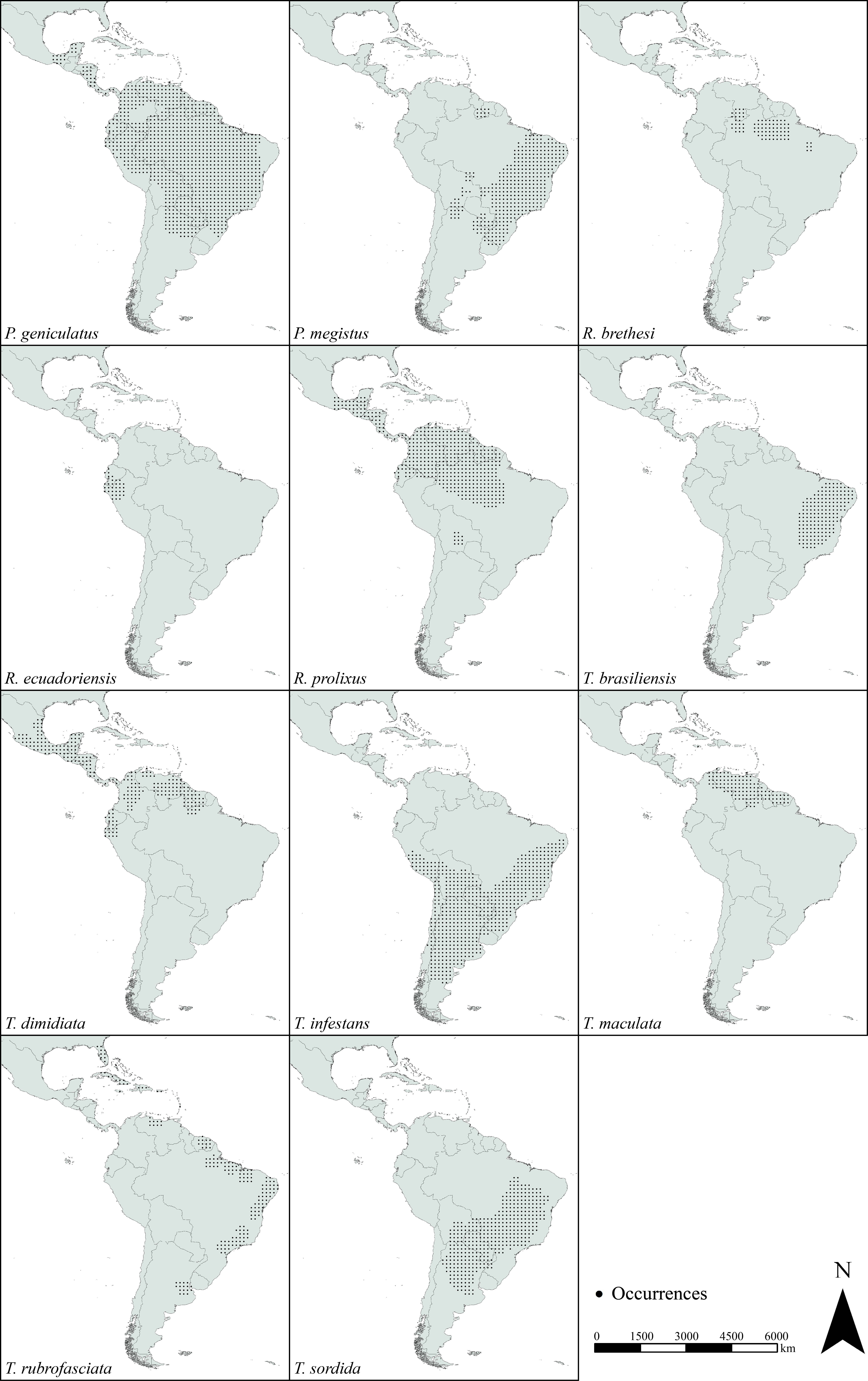

Supplement: Supplementary file 5. [file elife-52072-supp5.jpg]

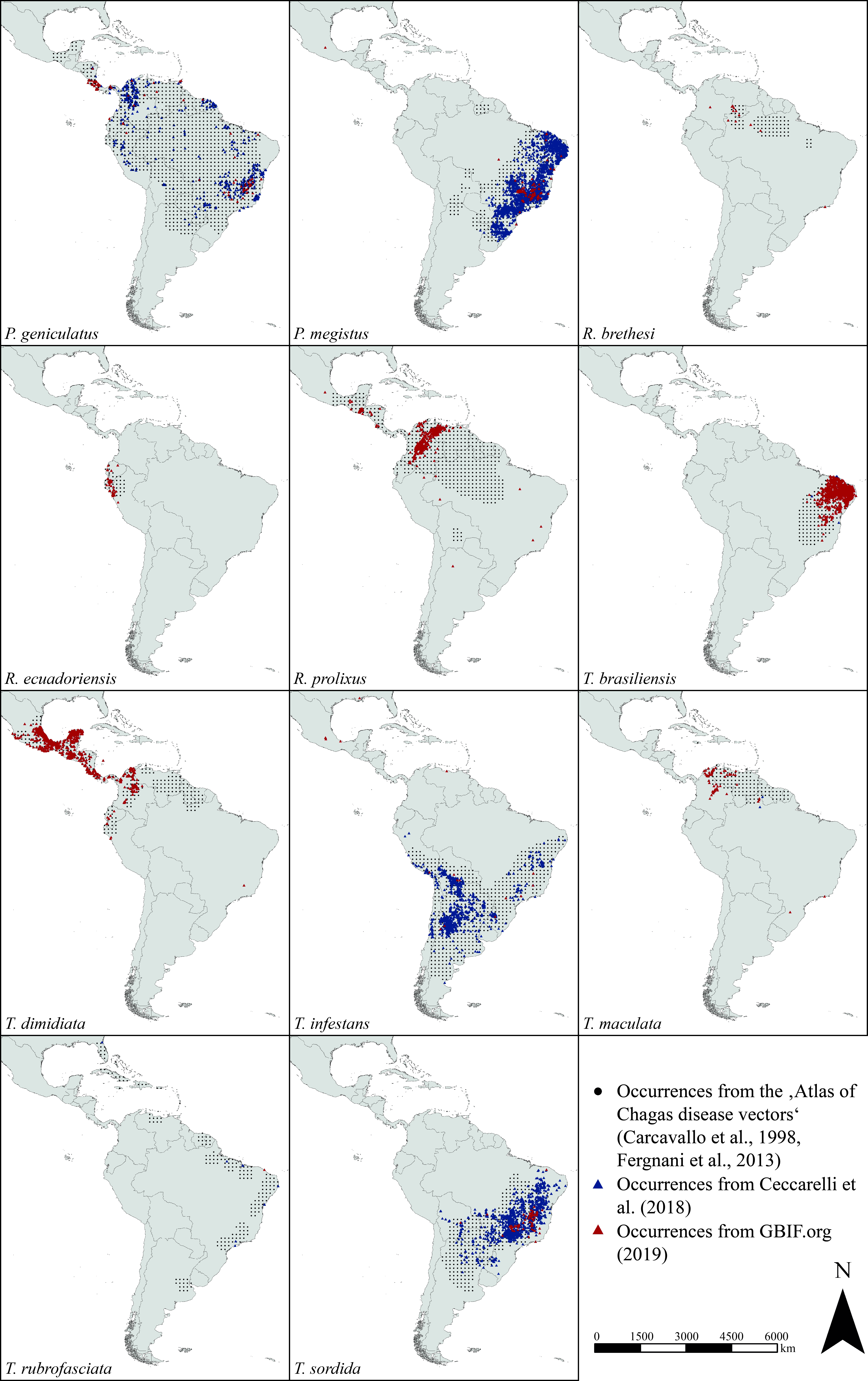

Supplement: Supplementary file 6. [file elife-52072-supp6.jpg]

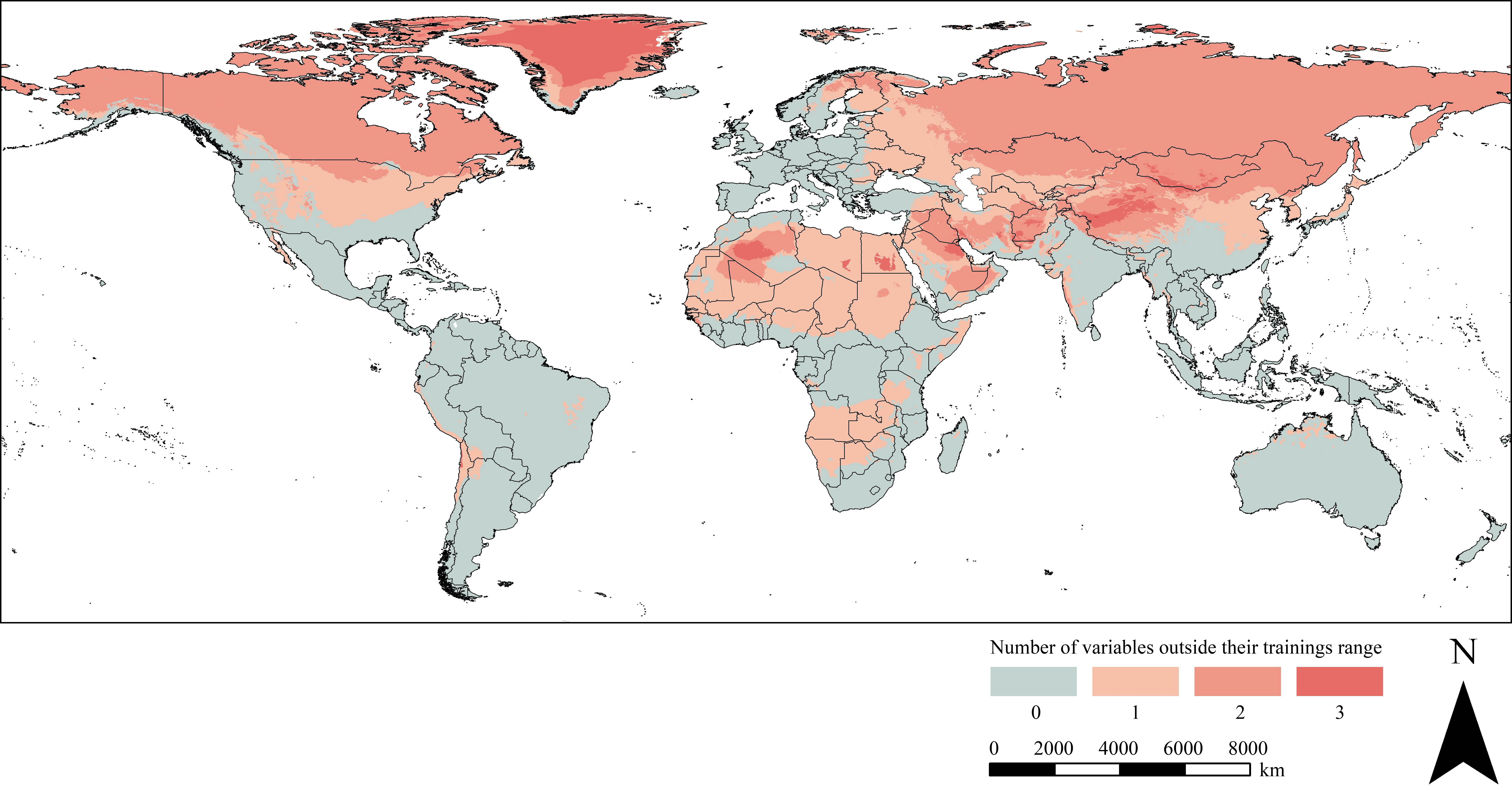

Supplement: Supplementary file 7. — The climatic suitability projections in these areas can be regarded as uncertain. [file elife-52072-supp7.jpg]
