# Supplementary material for: Modelling the climatic suitability of Chagas disease vectors on a global scale
Source: eLife. 2020 May 6;9:e52072. doi: 10.7554/eLife.52072 (PMC7237218; doi:10.7554/eLife.52072)
Supplement: Supplementary file 8. [file elife-52072-supp8.docx]

Supplementary File 8: Sensitivity and specificity metrics of all algorithms for all considered species.

| **Species** | **Sensitivity (se) and specificity (sp) values of all algorithms** | | | | | | | | | | | |
| --- | --- | --- | --- | --- | --- | --- | --- | --- | --- | --- | --- | --- |
|  | **ANN** | | **GAM** | | **GBM** | | **GLM** | | **MARS** | | **Maxent** | |
|  | se | sp | se | sp | se | sp | se | sp | se | sp | se | sp |
| *P. geniculatus* | 86.6 | 80 | 90.1 | 87.4 | 93.7 | 96.3 | 92.8 | 80.6 | 86 | 91.9 | 85.4 | 42.7 |
| *P. megistus* | 54.6 | 88.4 | 90.8 | 80 | 86.6 | 95.6 | 88.2 | 80.5 | 82.4 | 88.6 | 89.1 | 67.2 |
| *R. brethesi* | 96 | 84.4 | 92 | 90.2 | 88 | 90.9 | 88 | 90 | 92 | 87.8 | 88 | 83.8 |
| *R. ecuadoriensis* | 70 | 96.2 | 80 | 97.5 | 90 | 97.8 | 90 | 93.5 | 70 | 98.2 | 90 | 79.3 |
| *R. prolixus* | 93.7 | 85.7 | 94.4 | 87.2 | 96.5 | 91.3 | 97.9 | 80.5 | 94.4 | 86.4 | 82.5 | 73.2 |
| *T. brasiliensis* | 92.3 | 83.5 | 92.3 | 93.2 | 94.2 | 97.8 | 94.2 | 90.5 | 100 | 86.5 | 92.3 | 86 |
| *T. dimidiata* | 69.9 | 80.2 | 86.3 | 74.4 | 90.4 | 80 | 89.1 | 68 | 76.7 | 78.8 | 76.7 | 69.1 |
| *T. infestans* | 95.2 | 61.5 | 90.4 | 82.5 | 91.4 | 93.3 | 88.2 | 79 | 87.7 | 85.5 | 90.9 | 53.1 |
| *T. maculata* | 97.1 | 97.1 | 94.3 | 89.8 | 88.6 | 91.6 | 87.1 | 87.2 | 97.1 | 86.1 | 100 | 76.6 |
| *T. rubrofasciata* | 96.4 | 50.6 | 96.4 | 58.2 | 85.5 | 88.1 | 94.5 | 57.2 | 96.4 | 67.3 | 90.9 | 57 |
| *T. sordida* | 92.7 | 77.4 | 92.7 | 81.4 | 91.1 | 95.4 | 95.9 | 77.7 | 92.7 | 83.1 | 91.9 | 69.6 |
